# Predictive nature of IgM anti-α-glucose serum biomarker for relapse activity and EDSS progression in CIS patients: a BENEFIT study analysis

**DOI:** 10.1177/1352458511432327

**Published:** 2012-07

**Authors:** MS Freedman, C Metzig, L Kappos, CH Polman, G Edan, H-P Hartung, DH Miller, X Montalban, J Yarden, L Spector, E Fire, N Dotan, S Schwenke, V Lanius, R Sandbrink, C Pohl

**Affiliations:** 1 Multiple Sclerosis Research Clinic, The Ottawa Hospital – General Campus, Ottawa, Ontario, Canada; 2 Bayer Pharma AG, Berlin, Germany; 3 University Hospital, Basel, Switzerland; 4 Department of Neurology, Vrije Universiteit Medical Center, Amsterdam, The Netherlands; 5 Neurological Department, Clinique Neurologique, Rennes, France; 6 Department of Neurology, Heinrich-Heine-Universität, Düsseldorf, Germany; 7 Department of Neuroinflammation, Institute of Neurology, University College London, London, UK; 8 Unit of Clinical Neuroimmunology, Hospitals Vall d’Hebron, Barcelona, Spain; 9 Glycominds Ltd, Modi’in, Israel; 10 University Hospital of Bonn, Germany

**Keywords:** antibody, biomarker, clinically definite multiple sclerosis, clinically isolated syndrome, Expanded Disability Status Scale, multiple sclerosis, prognosis

## Abstract

**Background:**

Higher serum levels of at least one of a panel of four α-glucose IgM antibodies (gMS-Classifier1) in clinically isolated syndrome (CIS) patients are associated with imminent early relapse within 2 years.

**Objective:**

The objective of this study was to determine the prognostic value of gMS-Classifier1 in a large study cohort of CIS patients.

**Methods:**

The BEtaseron^®^ in Newly Emerging multiple sclerosis For Initial Treatment (BENEFIT) 5-year study was designed to evaluate the impact of early versus delayed interferon-β-1b (IFNβ-1b; Betaseron^®^) treatment in patients with a first event suggestive of multiple sclerosis (MS). Patients (*n* = 258, 61% of total) with a minimum of 2 ml baseline serum were eligible for the biomarker study. gMS-Classifier1 antibodies’ panel (anti-GAGA2, anti-GAGA3, anti-GAGA4 and anti-GAGA6) levels were measured blinded to clinical data. Subjects were classified as either ‘positive’ or ‘negative’ according to a classification rule.

**Results:**

gMS-Classifier1 was not predictive for the time to clinically definite MS or time to MS according to the revised McDonald’s criteria, but did significantly predict an increased risk for confirmed disability progression (log-rank test: *p* = 0.012).

**Conclusions:**

We could not confirm previous results that gMS-Classifier1 can predict early conversion to MS in CIS. However, raised titres of these antibodies may predict early disability progression in this patient population.

## Introduction

Multiple sclerosis (MS) is an inflammatory demyelinating disease of the central nervous system (CNS) with a highly active and unpredictable disease course. Clinically, it ranges from a benign course with little disability, to a fulminant, disabling disease. Initially, most MS patients enter a course with relapses and remissions, but some progress in the absence of attacks (primary progressive disease). Several purported biomarkers predict the course of MS, with clinical,^1–4^ immunologic^5^ and radiologic^6–8^ candidates identified. To date, only baseline brain MRI is somewhat predictive, with its greatest value being at the first presentation of clinically isolated syndrome (CIS).^1–10^ However, the variance in terms of MRI disease is so great that precise lesion measurement offers less value in predicting prognosis. Overall, an early MRI can predict with high certainty whether a patient will develop MS, but cannot accurately determine when this will occur or whether progression to disability is imminent.

With many different options for early MS treatment now available, there is an important medical need for a biomarker that, with some degree of certainty, provides information regarding the early disease course. Early promising candidate markers were antibodies to CNS antigens measured in serum or cerebrospinal fluid (CSF), such as those against myelin oligodendrocyte glycoprotein or myelin basic protein,^11^ but results are inconsistent.^12,13^ Subsequently, significantly higher levels of immunoglobulin M (IgM) anti-Glc(αl,4)Glc(α) antibodies (anti-GAGA4 antibodies) were found in relapsing–remitting MS (RRMS) patients when normalized for total IgM, than in patients with other neurologic diseases (OND).^14^ This was confirmed by another study in 739 MS patients, 65 with OND and 100 healthy controls.^15^ More recently, higher levels of at least one of a panel of anti-GAGA IgM antibodies (GAGA2, 3, 4, and 6), gMS-Classifier1, were more frequently observed in patients experiencing their first neurologic event and who were more likely to have a more rapid first relapse, which would establish the diagnosis of RRMS within 48 months (clinically definite MS [CDMS]), thus potentially serving as both a diagnostic and more importantly early prognostic marker for disease activity.^16^

The primary aim of this study was to confirm the hypothesis that positive gMS-Classifier1 could predict an earlier conversion to CDMS in a larger population of prospectively followed CIS patients, using data from the BEtaseron^®^ in Newly Emerging Multiple Sclerosis For Initial Treatment (BENEFIT) trial. Secondary aims were to evaluate whether these antibodies are associated with an increased risk of progression to MS diagnosis based on McDonald criteria (2005)^17^ and increased risk of confirmed Expanded Disability Status Scale (EDSS) progression along with some of the MRI measures of disease activity.

## Materials and methods

### Study population

The BENEFIT study was designed to evaluate the impact of early versus delayed interferon-β-1b (IFNβ-1b; Betaseron^®^, Bayer Pharma AG, Berlin, Germany) treatment in patients with a first event suggestive of MS, in the initial placebo-controlled phase patients with a minimum of two clinically silent MRI lesions and, thus, were randomized to either IFNβ-1b 250 µg (*n* = 292) or placebo (*n* = 176) subcutaneously every other day for 2 years, or until diagnosis of CDMS. All patients were then eligible to enter a prospectively planned, follow-up phase with open-label IFNβ-1b for a maximum of 5 years after randomization. Study details have been published elsewhere.^18^

### Blood sample analysis

Analyses were performed using baseline samples from BENEFIT obtained shortly before treatment initiation and up to 60 days after onset of the first MS event. Samples were shipped within 3 days of being drawn, under ambient conditions, then maintained at −20°C at the central laboratory until further analysis. A first-thaw process after the initial freeze was completed for this study.

### Measurement of anti-glycan IgM antibody levels and total IgM with glycan assay

Anti-glycan IgM antibodies measurements were only performed in patients with a minimum of 2 ml serum available at baseline representing a subcohort of 61% (286 patients) from the whole study. Baseline samples were analysed blindly. Levels of gMS-Classifier1 were determined in IgG-depleted samples by immunoassay (gMS^®^Pro EDSS test, Glycominds, Modi’in, Israel). In order to prevent IgM precipitation, samples were allowed to reach room temperature, then incubated at 37°C for 2 hours and mixed. IgM antibody measurement is stable under these conditions together with minimal freeze–thawing (two maximum). Micro-well plates with GAGA2, GAGA3, GAGA4 and GAGA6 antigens were prepared as described previously,^19^ anti-GAGA2, anti-GAGA3, anti-GAGA4 and anti-GAGA6 IgM assays were performed as described previously for GAGA4.^15^ Briefly, serum samples were diluted 1:1200, dispensed into the wells with GAGA antigens in duplicate, incubated for 180 min at 4°C, then washed with buffer. Bound antibodies were labelled with horseradish peroxidase-conjugated goat anti-human IgM type-specific antibody, washed and 3, 3’, 5, 5’-tetramethylbenzidine added for detection. After 30 min, the enzymatic reaction was stopped with 1% sulfuric acid solution, and optical density (OD) read at 450 nm (Victor 1420 plate reader; Wallac, Turku, Finland). Each plate included a five-point calibration curve and a blank. Results were reported in arbitrary units (U). The average OD of blank samples was subtracted from that of the patient samples before calculating the U.

### Statistical analysis

Subjects were classified as either gMS-Classifier1 ‘positive’ or ‘negative’ according to an adaptation of the classification rule described previously (see Supplementary Figure 1) that distinguished patients predicted to have a relapse within 2 years after their first event suggestive of MS.^16^ In the study in which the gMS-Classifier1 algorithm was constructed,^16^ the antibody levels were measured as relative fluorescence units using an immunofluorescence assay, and in the present study the antibody levels were reported using enzyme immunoassay units. Although the absolute values of the previous study’s cut-offs could not be applied directly to the present study, the method for determining the cut-off values was the same in both studies (details can be found in Supplementary Figure 1). The correlation between total IgM levels and gMS-Classifier1 antibodies was explored by Spearman’s correlation coefficient.

Performance characteristics such as the accuracy, sensitivity, specificity, positive and negative predictive value of this classifier (abbreviated as gMS-Classifier1 within this manuscript) for the prediction of an early CDMS diagnosis (<month 24) were calculated. In BENEFIT, as in the previous study,^16^ a diagnosis of CDMS was based on the occurrence of a second clinical event (i.e. first relapse).

In addition, the impact of gMS-Classifier1 on secondary objectives were evaluated in an analysis with respect to other key clinical and MRI variables in BENEFIT based on the data obtained up to year 5. Time to CDMS, McDonald MS and confirmed disability progression (defined as a 1.0-point increase on the EDSS confirmed over a minimum 6-month period) were tested by log-rank tests and Cox proportional hazards regression adjusted for age, sex, monofocal versus multifocal disease onset, steroid use at first event, presence of at least 1 gadolinium-enhanced lesion and ≥9 hyperintense T2 lesions at first event, total serum IgM (analysis for time to confirmed disability progression was performed with and without an additional adjustment for baseline EDSS). Annualized relapse rates were analysed with a generalized Poisson model, MRI variables (change from screen to month 60) by non-parametric analysis of covariance.

All analyses were performed in the total cohort with available gMS-Classifier1 data. Subgroup analyses on the classification of early CDMS by gMS-Classifier1 and the impact of the classifier on time-to-event outcomes were performed in patients randomized to IFNβ-1b using the integrated data of the double-blind and the follow-up study as well as in patients randomized to placebo using data of the placebo-controlled period only. Considering that patients entered the follow-up study when reaching CDMS (or after 2 years if no CDMS was diagnosed), the placebo subgroup was not analysed for time to confirmed EDSS progression, since patients developed disability progression only occasionally before that time point. *P*-values from secondary and subgroup analyses were not corrected for multiple comparisons. Therefore, they should be interpreted in an exploratory fashion.

## Results

### Patient characteristics

Of 286 samples with available gMS-Classifier1 measurements at the BENEFIT baseline visit, 109 patients were randomized to placebo and 177 to IFNβ-1b treatment. Regarding key screening and baseline characteristics, the overall BENEFIT study population and the investigated subcohort were similar (Table 1). A total of 255 patients of the 286 gave informed consent for the follow-up phase with open-label IFNβ-1b (95 initial placebo/160 initial IFNβ-1b) and were observed for up to 5 years.

**Table 1.** Screening and baseline characteristics of the overall cohort of BENEFIT and the subcohort investigated in this study.

	Overall BENEFIT study population (*n* = 468)	Investigated subcohort of BENEFIT (*n* = 286)
Gender (female), % (*n*)	70.7% (331)	73% (209)
Mean/median age at study start,	30.7/30	31.2/31
standard deviation	7.4	7.4
min–max	18–45	18–45
Patients with steroid use at time of the first event, % (*n*)	70.9% (332)	73.8% (211)
Monofocal onset, % (*n*)	52.6% (246)	53.8% (154)
Gadolinium-enhanced lesions, % (*n*)	42.7% (198/464)	42.3% (120)
Mean/median number of T2-lesions	28.3/17.0	30.5/19.0
standard deviation	30.6	30.8
min–max	2-194	2-194
Randomized treatment, % initial IFNβ-1b (*n*)	62.4% (292)	61.9% (177)

BENEFIT, BEtaseron^®^ in Newly Emerging multiple sclerosis For Initial Treatment; IFNβ-1b, interferonβ-1b.

Descriptive statistics for the anti-glycan IgM antibody measurements in the baseline serum samples are shown in the supplementary data.

Spearman’s correlation coefficient for the gMS-Classifier1 antibodies with the total IgM level were 0.39 for anti-GAGA2, 0.50 for anti-GAGA3, 0.38 for anti-GAGA4 and 0.57 for anti-GAGA6.

### gMS-Classifier1 performance characteristics for the primary outcome, CDMS prognosis

For the evaluation of gMS-Classifier1, a patient was defined as having an early CDMS diagnosis (<month 24), if CDMS was diagnosed up to day 692, and as not having an early CDMS diagnosis if the patient was still observed on day 692 but CDMS was not diagnosed. Seventeen patients who discontinued the study before day 692 without having a CDMS diagnosis were censored; therefore, 269 patients remained for evaluation of the classifier with respect to predicting early MS.

Based on estimated means and standard deviations for the antibody measurements, the following classification rule was derived: a patient was classified as ‘positive’ for gMS-Classifier1, if anti-GAGA2 >148.8 EIA units, anti-GAGA3 >164.6 EIA units, anti-GAGA4 >133.6 EIA units or anti-GAGA6 >168.1 EIA units (see Supplementary Table 1 and Supplementary Figure 1). Of 269 patients available for classifier evaluation, 50 were ‘positive’. Of 89 patients with a CDMS diagnosis up to month 24, 19 were correctly predicted as ‘positive’ (Table 2 shows the 2 × 2 table based on this classification).

**Table 2.** Classification table for gMS-Classifier1 and the CDMS status after 24 months.

Observed gMS-Classifier1	Early CDMS (≤24 months) diagnosed		Sum
	Yes	No	
Positive, i.e. early CDMS	19	31	50
Negative, i.e. no early CDMS	70	149	219
Sum	89	180	269

CDMS, clinically definite multiple sclerosis.

**Figure 1.**
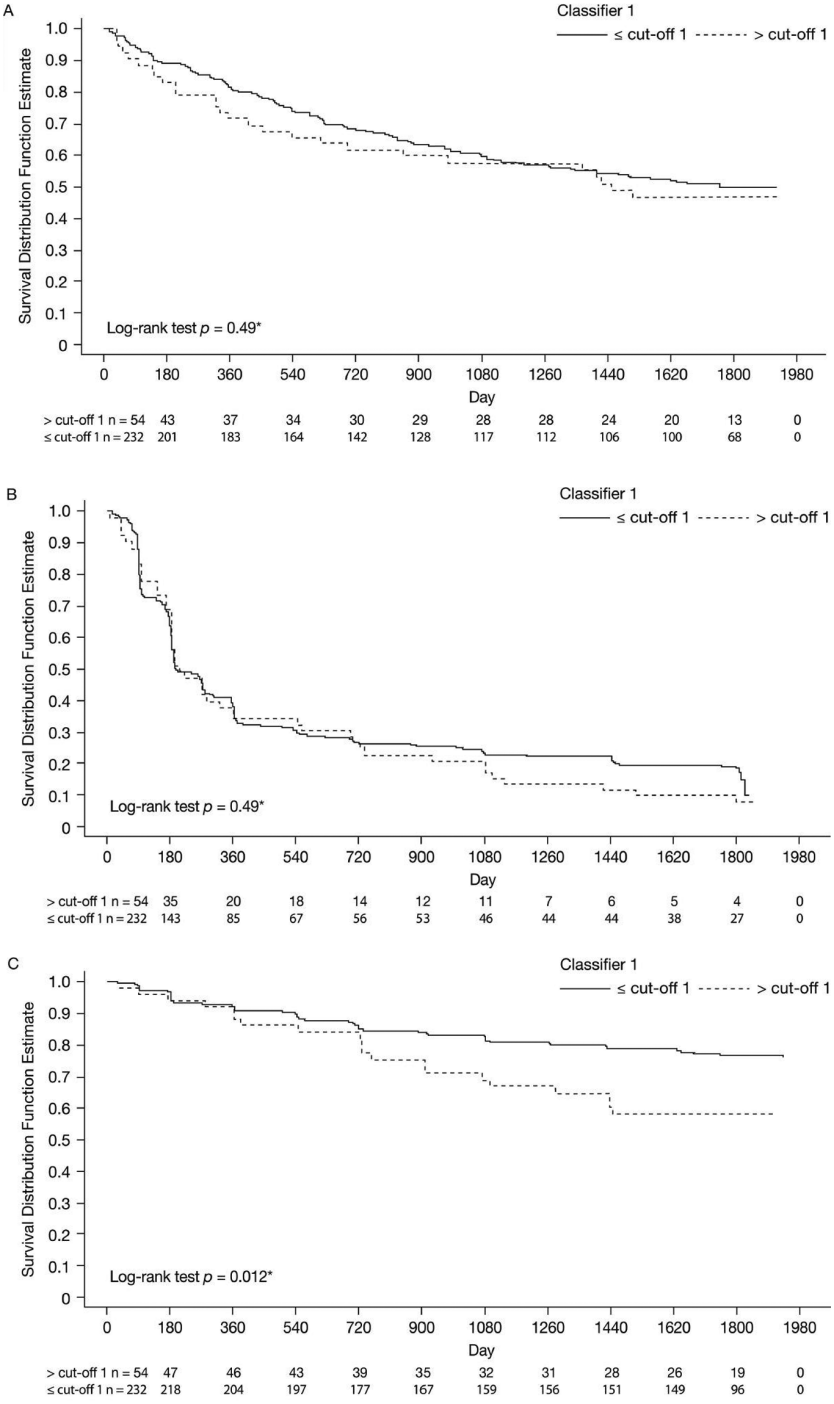
Kaplan–Meier curves for gMS-Classifier1 positive versus negative patients with respect to (A) time to clinically definite multiple sclerosis, (B) time to McDonald MS and (C) time to confirmed Expanded Disability Status Scale (EDSS) progression. *Log-rank test *p*-value for comparison of gMS-Classifier1 groups with respect to time-to-event variables. Analyses performed using sera set A.

From these data, gMS-Classifier1 showed an estimated sensitivity of 21.4% (95% confidence interval [CI]: 12.8–29.9%) when a high specificity of >80% (point estimate: 82.8%; 95% CI: 77.3–88.3%) was required for the prediction of early CDMS (accuracy: 62.5%, 56.7–68.2%; positive predictive value: 38.0%, 95% CI 24.6–51.5%). These test performance characteristics were very similar in the subgroups initially randomized to IFNβ-1b or during the placebo period (data not shown). These data show that gMS-Classifier1 has no significant predictive value regarding the prognosis of CDMS (Figure 1A) for up to 5 years after CIS.

### Secondary aims: analysis of the impact of the gMS-Classifier1 on other disease outcomes

Table 3 shows the results of the analyses of the secondary time-to-event variables over the observation period of up to 5 years (286 patients with antibody data of which 54 were positive for gMS-Classifier1 were used for these analyses).

**Table 3.** Results for comparison of the gMS-Classifier1 groups with respect to time-to-event variables.

Time-to-event outcome	Log-rank test	Cox proportional hazard regression^a^		
	*p*-value	Hazard ratio	95% CI	*p*-value
Time to CDMS	0.4925	1.26	0.82–1.94	0.2951
Time to McDonald MS	0.4552	1.10	0.80–1.51	0.5768
Time to confirmed EDSS progression	0.01	2.05	1.20–3.51	0.009
Time to confirmed EDSS progression (adjusted^b^)		1.63	0.95–2.80	0.07

a With adjustment for baseline covariates: age, sex, onset of disease (multifocal versus monofocal), steroid use at the first event, treatment, presence of gadolinium-enhanced lesions (no versus at least one lesion) and number of hyperintense T2 lesions (<9 versus ≥9) and total IgM (in mg/ml).

b With additional adjustment for baseline EDSS score.

CI, confidence interval; CDMS, clinically definite multiple sclerosis; EDSS, Expanded Disability Status Scale; MS, multiple sclerosis.

These data show that gMS-Classifier1 has no significant predictive value regarding the conversion to McDonald (2005) MS (Figure 1B) for up to 5 years after CIS. These results were similar for the analyses in the subgroup of patients initially randomized to IFNβ-1b or during the placebo period (data not shown). However, the risk of having a confirmed EDSS progression for patients classified as ‘positive’ was about twice the risk for patients classified as ‘negative’ (hazard ratio = 2.05 [95% CI: 1.2–3.5], *p* = 0.009). A lower increase in risk for confirmed EDSS progression that did not reach the level of statistical significance that was observed in the Cox model if baseline EDSS was considered as an additional covariate (1.63 [95% CI: 0.95–2.8], *p* = 0.07). At year 5, according to the Kaplan–Meier estimates, 23.3% of CIS patients classified as negative for gMS-Classifier1 developed confirmed EDSS progression versus 41.8% classified as positive (Figure 1C). If analyses were restricted to patients initially randomized to IFNβ-1b statistical significance was lost although there was still a higher risk for gMS-Classifier1 positive patients to experience confirmed EDSS progression (Kaplan–Meier estimates at year 5 in gMS-Classifier1 negative versus positive patients: 21% versus 34%, log-rank test: *p* = 0.16).

### Additional findings

Based on the data obtained up to year 5, the annualized relapse rates in both classifier groups were comparable (*p* = 0.13). With respect to MRI, no difference between the classification groups was observed for change in T2 hyperintense lesion volume (*p* = 0.31), change in T1 hypointense lesion volume (*p* = 0.20) and percentage change in brain volume (*p* = 0.75).

## Discussion

The BENEFIT study afforded a prospectively collected database of patients followed up to 5 years after presenting with their first event of MS to test the potential prognostic value of a putative serum biomarker taken at baseline. Although a smaller retrospective study suggested that gMS-Classifier1 based on measuring a panel of four anti-glycan IgM antibodies might predict an early (<24 months) relapse signifying CDMS, we did not find any evidence supporting this classifier. In the previous hypothesis-generating study,^16^ the same classifier predicted early relapse with a sensitivity of 38% at a specificity of 88% (*n* = 100; χ^2^-test for early relapse activity versus positive gMS- Classifier1 findings: *p* = 0.01, positive predictive value of gMS-Classifier1: 81%). In our study of 286 patients followed for up to 5 years after the first event suggestive of MS, sensitivity of this gMS-Classifier1 to predict a diagnosis of clinically definite MS within 2 years dropped to 21.3% at a specificity of 82.8%, yielding a much lower positive predictive value of 38.0%. Moreover, there was no statistically significant increase in the risk for CDMS over the observation period in patients positive for gMS-Classifier1. This result was the same in patients initially randomized to placebo or IFNβ-1b for up to 2 years. One possible explanation for the difference in behaviour of the classifier could be that in the previous study,^16^ all patients had MS (i.e. experienced events satisfying the diagnostic criteria for MS) and the median time to CDMS was 20 months. In the present study, MS diagnosis occurred in less than 50% of the cohort, and the cohort did not reach median time to CDMS with only 46.9% having a second relapse even after 60 months. Furthermore, whereas patients in the previous study were recruited from only two centres in Western Europe and Canada, our study recruited patients from 98 sites in Europe and Canada. Therefore, the study population of the previous trial was more homogeneous and enriched for clinical disease activity, possibly improving the chance of detecting prognostic effects of the investigated biomarker. Nevertheless, the negative result of our study does not support the use of the evaluated gMS-Classifier1 for predicting early relapse activity in patients with a first event suggestive of MS. This negative result was also corroborated by the lack of any effect of gMS-Classifier1 on MRI outcomes. Therefore, apart from the clinical presentation at the time of the first event, the presence of oligoclonal bands, and the distribution and number of CNS lesions on MRI are still the best predictors of future clinical and MRI activity in patients with early MS.^6–8,20,21^

Relapses, MRI activity and disease progression are the three most evaluated outcomes in MS. It is still unclear how these outcomes are related; new MRI activity often correlates with relapse rates, but neither correlates well with disease progression. Indeed, it is uncertain whether MRI activity or relapses even affect disease progression, once disease progression is evident.^22–24^ It was, therefore, interesting that we observed a significant prognostic value of the evaluated gMS-Classifier1 for the accumulation of clinical disability. We found that patients positive for one of four anti-glycan IgM antibodies had nearly twice the risk of progressing in the 5 years of this study. An increase in the risk for confirmed EDSS progression was even seen in gMS-Classifier1 positive patients treated with IFNβ-1b from the first event (i.e. the early treatment cohort), although this was no longer statistically significant possibly due to the lower number of patients contributing to this analysis (177 instead of 286).

Most EDSS progression in early MS is due to relapse-related residual deficit.^25^ Some attacks are mild with easily reversible deficits, while others are more serious and leave residual deficits. A classifier predicting faster disability progression in RRMS patients may therefore indicate more serious attacks.^25,26^ Since IFNβ-1b has a known treatment effect at reducing both relapses and progression,^18,24,27^ this study cohort would have experienced fewer relapses and progressions than an untreated study population, which would impact the classifier by increasing the number of false negatives in serum collected prior to treatment (i.e. the classifier might have had a better performance only the treatment served to prevent the events that were predicted to occur) and it is unlikely that any current cohort of early MS patients are left untreated. When baseline EDSS was used as a covariant (Table 3), the classifier lost its significance for predicting early progression, probably because baseline EDSS itself is predictive for early disease progression,^28^ a finding underlining the need to confirm our exploratory results on EDSS progression in an independent study.

Any biomarker that predicts early disease progression could be perceived as more useful to the clinician than one indicating a high risk for relapse. If our findings on EDSS progression can be confirmed, gMS-Classifier1 may be used to differentiate between patients who will have relatively severe residual deficits from attacks to those with reversible deficits. All current first-line, disease-modifying medications reduce the risk for subsequent attacks and lower MRI activity, and have shown benefit in patients at CIS,^17,29–31^ so it is unlikely that CIS patients would not be treated with one of these agents, obviating the need for a baseline biomarker predicting early relapse. Conversely, much discussion revolves around the potential use of ‘induction’ versus ‘escalation’ treatment for certain patients, especially those deemed to be at very high risk for early and imminent disease progression.^32^ We now have several very effective agents that control disease activity (e.g. natalizumab) but they also carry higher toxicity risks. It is difficult to predict who will be more likely to do poorly and progress, although some early prognostic markers have helped.^33,34^ The availability of a biomarker that can accurately predict who might be at high risk of early disease progression, in addition to the known clinical prognostic markers, could help steer the choice of therapy towards a more aggressive course of treatment.

The identification of prognostic markers for disability progression in MS is notoriously difficult, since fixed disability evolves slowly over time in most patients, necessitating very long observation periods to detect relevant changes. In this respect, it is a strength of the present study that an observational period of 5 years was available in a substantial number of patients. However, even over this observation time, progression of clinical disability was a rare event given a CIS cohort treated with IFNβ-1b from the first event or up to 2 years at the latest after disease onset.^18^ Not all patients present for therapy at this early stage. Some already have a diagnosis of MS or CDMS (at least two distinct clinical attacks), whereas others may have done well initially on first-line treatments, but disease breakthrough warrants a change in treatment. It is therefore very important that the utility of a classifier such as anti-glycan IgM antibody measurement be confirmed in other studies examining different stages of MS as well as testing its ability to change over time in response to treatment and for monitoring disease activity to predict breakthrough.

## References

[1] McAlpine D The benign form of multiple sclerosis. A study based on 241 cases seen within three years of onset and followed up until the tenth year or more of the disease. Brain 1961; 84: 186–203 13773723 10.1093/brain/84.2.186

[2] Weinshenker BG Bass B Rice GP Noseworthy J Carriere W Baskerville J The natural history of multiple sclerosis: a geographically based study. 2. Predictive value of the early clinical course. Brain 1989; 112: 1419–1428 2597989 10.1093/brain/112.6.1419

[3] Runmarker B Andersen O Prognostic factors in a multiple sclerosis incidence cohort with twenty five years of follow-up. Brain 1993; 116: 117–134 8453453 10.1093/brain/116.1.117

[4] Achiron A Barak Y Multiple sclerosis from probable to definite diagnosis: a 7 year prospective study. Arch Neurol 2000; 57: 974–979 10891979 10.1001/archneur.57.7.974

[5] Galboiz Y Miller A lmmunological indicators of disease activity and prognosis in multiple sclerosis. Curr Opin Neurol 2002; 15: 233–237 12045718 10.1097/00019052-200206000-00002

[6] O’Riordan JI Thompson AJ Kingsley DPE MacManus DG Kendall BE Rudge P The prognostic value of brain MRI in clinically isolated syndromes of the CNS: a 10-year follow-up. Brain 1998; 121: 495–503 9549525 10.1093/brain/121.3.495

[7] Brex PA Ciccarelli O O’Riordan JI Sailer M Thompson AJ Miller DH A longitudinal study of abnormalities on MRI and disability from multiple sclerosis. N Engl J Med 2002; 346: 158–164 11796849 10.1056/NEJMoa011341

[8] Dalton CM Brex PA Miszkiel KA Hickman SJ MacManus DG Plant GT Application of the new McDonald criteria to patients with clinically isolated syndromes suggestive of multiple sclerosis. Ann Neurol 2002; 52: 47–53 12112046 10.1002/ana.10240

[9] Fisniku LK Brex PA Altmann DR Miszkiel KA Benton CE Lanyon R Disability and T2 MRI lesions: a 20-year follow-up of patients with relapse onset of multiple sclerosis. Brain 2008; 131: 808–817 18234696 10.1093/brain/awm329

[10] Tintoré M Rovira A Río J Nos C Grivé E Téllez N Baseline MRI predicts future attacks and disability in clinically isolated syndromes. Neurology 2006; 67: 968–972 17000962 10.1212/01.wnl.0000237354.10144.ec

[11] Berger T Rubner P Schautzer F Egg R Ulmer H Mayringer I Antimyelin antibodies as a predictor of clinically definite multiple sclerosis after a first demyelinating event. N Engl J Med 2003; 349: 139–145 12853586 10.1056/NEJMoa022328

[12] Kuhle J Pohl C Mehling M Edan G Freedman MS Hartung HP Lack of association between antimyelin antibodies and progression to multiple sclerosis. N Engl J Med 2007; 356: 371–378 17251533 10.1056/NEJMoa063602

[13] Pelayo R Tintoré M Montalban X Rovira A Espejo C Reindl M Antimyelin antibodies with no progression to multiple sclerosis. N Engl J Med 2007; 356: 426–428 17251547 10.1056/NEJMc062467

[14] Schwarz M Spector L Gortler M Weisshaus O Glass-Marmor L Karni A Serum anti-Glc(alphal,4)Glc(alpha) antibodies as a biomarker for relapsing-remitting multiple sclerosis. J Neurol Sci 2006; 244: 59–68 16480743 10.1016/j.jns.2005.12.006

[15] Brettschneider J Jaskowski TD Tumani H Abdul S Husebye D Seraj H Serum anti-GAGA4 IgM antibodies differentiate relapsing remitting and secondary progressive multiple sclerosis from primary progressive multiple sclerosis and other neurological diseases. J Neuroimmunol 2009; 217: 95–101 19879655 10.1016/j.jneuroim.2009.07.017

[16] Freedman MS Laks J Dotan N Altstock RT Dukler A Sindic CJ Anti-alpha-glucose-based glycan IgM antibodies predict relapse activity in multiple sclerosis after the first neurological event. Mult Scler 2009; 15: 422–430 19324980 10.1177/1352458508101944 PMC2850589

[17] Polman CH Reingold SC Edan G Filippi M Hartung HP Kappos L Diagnostic criteria for multiple sclerosis: 2005 revisions to the “McDonald Criteria”. Ann Neurol 2005; 58: 840-846 16283615 10.1002/ana.20703

[18] Kappos L Freedman MS Polman CH Edan G Hartung HP Miller DH BENEFIT Study Group. Long-term effect of early treatment with interferon beta 1b after a first clinical event suggestive of multiple sclerosis: 5-year active treatment extension of the phase 3 BENEFIT trial. Lancet Neurol 2009; 8: 987–997 19748319 10.1016/S1474-4422(09)70237-6

[19] Schwarz M Spector L Gargir A Shtevi A Gortler M Altstock RT A new kind of carbohydrate array, its use for profiling antiglycan antibodies, and the discovery of a novel human cellulose-binding antibody. Glycobiology 2003; 13: 749–754 12851287 10.1093/glycob/cwg091

[20] Masjuan J Alvarez-Cermeño JC García-Barragán N Díaz-Sánchez M Espiño M Sádaba MC Clinically isolated syndromes: a new oligoclonal band test accurately predicts conversion to MS. Neurology 2006; 66: 576–578 16505315 10.1212/01.wnl.0000198253.35119.83

[21] Tintoré M Rovira A Río J Tur C Pelayo R Nos C Do oligoclonal bands add information to MRI in first attacks of multiple sclerosis? Neurology 2008; 70: 1079–1083 17881717 10.1212/01.wnl.0000280576.73609.c6

[22] Confavreux C Vukusic S Moreau T Adeleine P Relapses and progression of disability in multiple sclerosis. New Engl J Med 2000; 343: 1430–1438 11078767 10.1056/NEJM200011163432001

[23] Confavreux C Vukusic S Adeleine P Early clinical predictors and progression of irreversible disability in multiple sclerosis: an amnesic process. Brain 2003; 126: 770–782 12615637 10.1093/brain/awg081

[24] Scalfari A Neuhaus A Degenhardt A Rice GP Muraro PA Daumer M The natural history of multiple sclerosis: a geographically based study 10: relapses and long-term disability. Brain 2010; 133: 1914–1929 20534650 10.1093/brain/awq118 PMC2892939

[25] Lublin FD Baier M Cutter G Effect of relapses on development of residual deficit in multiple sclerosis. Neurology 2003; 61: 1528-1532 14663037 10.1212/01.wnl.0000096175.39831.21

[26] Lublin FD The incomplete nature of multiple sclerosis relapse resolution. J Neurol Sci 2007; 256: S14-S18 17337274 10.1016/j.jns.2007.01.062

[27] Kappos L Polman CH Freedman MS Edan G Hartung HP Miller DH Treatment with interferon beta 1b delays conversion to clinically definite and McDonald MS in patients with clinically isolated syndromes. Neurology 2006; 67: 1242–1249 16914693 10.1212/01.wnl.0000237641.33768.8d

[28] Degenhardt A. Ramagopalan SV Scalfari A Ebers GC Clinical prognostic factors in multiple sclerosis: a natural history review. Nat Rev Neurol 2009; 5: 672–682 19953117 10.1038/nrneurol.2009.178

[29] Comi G Filippi M Barkhof F Durelli L Edan G Fernández O Effect of early interferon treatment on conversion to definite multiple sclerosis: a randomised study. Lancet 2001; 357: 1576–1582 11377645 10.1016/s0140-6736(00)04725-5

[30] Jacobs LD Beck RW Simon JH Kinkel RP Brownscheidle CM Murray TJ Intramuscular interferon beta-1a therapy initiated during a first demyelinating event in multiple sclerosis. CHAMPS Study Group. N Engl J Med 2000; 343: 898–904 11006365 10.1056/NEJM200009283431301

[31] Comi G Martinelli V Rodegher M Durelli L Edan G Fernández O; PreCISe study group. Effect of glatiramer acetate on conversion to clinically definite multiple sclerosis in patients with clinically isolated syndrome (PreCISe study): a randomised, double-blind, placebo-controlled trial. Lancet 2009; 374: 1503–1511 19815268 10.1016/S0140-6736(09)61259-9

[32] Freedman MS Induction vs. escalation of therapy for relapsing multiple sclerosis: the evidence. Neurol Sci 2008; 29: S47–S51 18690508 10.1007/s10072-008-0953-y

[33] Miller JR The importance of early diagnosis of multiple sclerosis. J Manag Care Pharm 2004; 10: S4–S11 15253684

[34] Kantarci OH Weinshenker BG Prognostic factors in multiple sclerosis. In Cook SD (ed.) Handbook of Multiple Sclerosis, third edition New York: Marcel Dekker, 2001, pp. 449–463

